# Efficacy and Safety of Asfotase Alfa in Infants and Young Children With Hypophosphatasia: A Phase 2 Open-Label Study

**DOI:** 10.1210/jc.2018-02335

**Published:** 2019-02-27

**Authors:** Christine E Hofmann, Paul Harmatz, Jerry Vockley, Wolfgang Högler, Hideki Nakayama, Nick Bishop, Gabriel Á Martos-Moreno, Scott Moseley, Kenji P Fujita, Johannes Liese, Cheryl Rockman-Greenberg, ENB-010-10 Study Group

**Affiliations:** 1 University Children’s Hospital, University of Würzburg, Würzburg, Germany; 2 University of California San Francisco Benioff Children’s Hospital Oakland, Oakland, California; 3 University of Pittsburgh School of Medicine, Pittsburgh, Pennsylvania; 4 University of Pittsburgh, Graduate School of Public Health, Pittsburgh, Pennsylvania; 5 Department of Paediatrics and Adolescent Medicine, Johannes Kepler University, Linz, Austria; 6 Institute of Metabolism and Systems Research, University of Birmingham, Birmingham, United Kingdom; 7 Fukuoka Higashi Medical Center, Fukuoka, Japan; 8 Sheffield Children’s Hospital, Sheffield, United Kingdom; 9 University of Sheffield, Sheffield, United Kingdom; 10 Hospital Infantil Universitario Niño Jesús, IIS La Princesa, Madrid, Spain; 11 Universidad Autónoma de Madrid, Madrid, Spain; 12 Centro de Investigación Biomédica en Red de la Fisiopatología de la Obesidad y Nutrición, Instituto de Salud Carlos III, Madrid, Spain; 13 Alexion Pharmaceuticals, Inc., Boston, Massachusetts; 14 Department of Pediatrics, University Hospital of Würzburg, Würzburg, Germany; 15 Children’s Hospital Research Institute of Manitoba, Winnipeg, Manitoba, Canada; 16 University of Manitoba, Winnipeg, Manitoba, Canada

## Abstract

**Context:**

Long-term data on enzyme replacement treatment of hypophosphatasia (HPP) are limited.

**Objective:**

To evaluate efficacy and safety of asfotase alfa in patients aged ≤5 years with HPP followed for up to 6 years.

**Design:**

Phase 2 open-label study (July 2010 to September 2016).

**Setting:**

Twenty-two sites; 12 countries.

**Participants:**

Sixty-nine patients [median (range) age: 16.0 (0.02 to 72) months] with severe HPP and sign/symptom onset before age 6 months.

**Intervention:**

Asfotase alfa 2 mg/kg three times/week or 1 mg/kg six times/week subcutaneously.

**Main Outcome Measures:**

Primary efficacy measure: Radiographic Global Impression of Change (RGI-C) score [−3 (severe worsening) to +3 (complete/near-complete healing)]. Additional outcome measures: respiratory status, growth, and safety. *Post hoc* analysis: characteristics of radiographic responders vs nonresponders at Year 1 (RGI-C: ≥+2 vs <+2).

**Results:**

During median (minimum, maximum) 2.3 (0.02, 5.8) years of treatment, RGI-C scores improved significantly at Month 6 [+2.0 (−1.7, +3.0)], Year 1 [+2.0 (−2.3, +3.0)], and Last Assessment [+2.3 (−2.7, +3.0); *P* < 0.0001 all]. Of 24 patients requiring respiratory support at Baseline, 11 (46%) no longer needed support. Height/weight *z* scores generally increased. Nine patients died (13%). All patients experienced at least one adverse event; pyrexia was most common. Compared with responders [n = 50 (72%)], nonresponders [n = 19 (28%)] had more severe disease at Baseline and a higher rate of neutralizing antibodies (NAbs) at Last Assessment.

**Conclusions:**

Most infants/young children given asfotase alfa showed early radiographic and clinical improvement sustained up to 6 years; radiographic nonresponders had more severe disease and more frequent NAbs at Last Assessment.

## Teaser

Most infants and young children with hypophosphatasia, treated with asfotase alfa, showed improved skeletal manifestations, respiratory function, and growth within 1 year, maintained up to 6 years.

Hypophosphatasia (HPP) is the rare, inherited, systemic, metabolic disease characterized by low activity of the tissue-nonspecific isoenzyme of alkaline phosphatase (TNSALP), which leads to extracellular accumulation of its substrates, mainly inorganic pyrophosphate (PPi) and pyridoxal 5′-phosphate (PLP) (1–4). Increased extracellular levels of PPi inhibit bone mineralization and lead to impaired skeletal mineralization in affected patients and additional rickets-like deformities in infants and children (2, 4). Reduced dephosphorylation of PLP, the circulating form of vitamin B6, by TNSALP has been associated with vitamin B6-responsive seizures in infants with HPP (3, 5).

Clinical presentation of HPP varies with age at onset, from *in utero* to adulthood (2, 6). Characteristic signs, symptoms, and complications of perinatal and infantile HPP that are potentially life threatening include respiratory failure, vitamin B6-responsive seizures, chest deformity, and craniosynostosis; other manifestations include severe hypercalcemia, nephrocalcinosis, poor growth, osteomalacia, and bowing of the long bones (2, 5, 7–11). Historically, patients with perinatal and infantile HPP have 58% to 100% mortality during the first year of life (12–14). The most common causes of death among infants with HPP is respiratory failure secondary chest deformity and pulmonary hypoplasia (12, 15).

Asfotase alfa (Strensiq^®^; Alexion Pharmaceuticals, Inc., Boston, MA) is a human recombinant TNSALP enzyme-replacement therapy approved for patients with pediatric-onset HPP (16). In an open-label study of 11 infants and young children (aged ≤3 years) with life-threatening HPP, treatment with asfotase alfa for up to 7 years improved HPP-related skeletal abnormalities seen on radiograph, respiratory function, growth, and cognitive and motor function (17, 18). Here we report the long-term safety and efficacy of asfotase alfa of infants and children aged ≤5 years with manifestations of HPP before age 6 months.

## Materials and Methods

### Patients

Children aged ≤5 years with signs or symptoms of HPP before age 6 months were eligible for enrollment if they had a documented diagnosis of HPP. Diagnosis of HPP required the following: total serum alkaline phosphatase (ALP) activity below the lower limit of normal for age, plasma PLP above the upper limit of normal (unless the patient was receiving pyridoxine for seizures), radiographic evidence of HPP (flared and frayed metaphyses; widened growth plates; areas of radiolucency or sclerosis; or severe, generalized osteopenia), and at least two HPP-related findings (history or presence of nontraumatic postnatal fracture and/or delayed fracture healing, nephrocalcinosis or history of elevated serum calcium, functional craniosynostosis, respiratory compromise or rachitic chest deformity, vitamin B6-responsive seizures, or failure to thrive). Exclusion criteria were serum calcium or phosphate levels below the normal range, serum 25(OH) vitamin D levels <20 ng/mL, current evidence of a treatable form of rickets, prior treatment with bisphosphonates, investigational drug treatment within 1 month, or current enrollment in any other study involving a new drug, device, or treatment of HPP.

The study complied with the Declaration of Helsinki and International Conference on Harmonization Guideline for Good Clinical Practice and with national, state, and local laws of pertinent regulatory authorities. The protocol was approved by each site’s Institutional Review Board/independent Ethics Committee, and written, informed consent was obtained for all patients from a parent(s) or guardian(s).

### Study design

In this open-label, multicenter, single-arm, multinational study [ClinicalTrials.gov NCT01176266; European Union Drug Regulating Authorities Clinical Trials (EudraCT) 2010-019850-42], eligible patients received a total subcutaneous dose of 6 mg/kg/week of asfotase alfa, administered as 1 mg/kg six times per week or 2 mg/kg three times per week (maximum volume: 1 mL asfotase alfa per injection). Dose adjustments were allowed at the investigator’s discretion to account for changes in body weight or in consultation with the medical monitor for safety concerns or lack of efficacy. The maximum dose permitted was 40 mg per injection or 9 mg/kg/week in Australia, France, Germany, Italy, Saudi Arabia, Spain, and the United Kingdom per protocol amendment; no dose restrictions were applied in Canada, Japan, Russia, Turkey, or the United States. The initial dose of asfotase alfa was administered at the study site during the Baseline visit; post-Baseline injections could be administered at home by a parent, legal guardian, or designee after adequate training. With each injection, the designated individual was required to complete a worksheet regarding the patient’s health condition, any new medications, and details of the injection. Study visits were scheduled at Weeks 3 and 6; Months 3, 6, 9, 12, 15, 18, and 24; and every 6 months thereafter until the end-of-study assessment (Month 48 in patients enrolled in the United Kingdom and final, every 6-month assessment in other countries). Patients were enrolled starting 22 July 2010, and the last patient completed the study 26 September 2016.

### Outcomes measures

#### Primary efficacy measure

The primary efficacy measure was improvement of HPP-related skeletal manifestations at Week 24 (Month 6) and Week 48 (Year 1) of treatment, as measured on the Radiographic Global Impression of Change (RGI-C) scale (19). The RGI-C (19) is a validated seven-point scale that assesses changes from Baseline in HPP-related skeletal abnormalities: −3 = severe worsening, −2 = moderate worsening, −1 = minimal worsening, 0 = no change, +1 = minimal healing, +2 = substantial healing, and +3 = complete or near-complete healing. Radiographs of the chest, bilateral wrists, and bilateral knees were reviewed by three independent pediatric radiologists, and comparisons with Baseline were scored. The mean RGI-C score for each patient at each time point was calculated from available scores. The radiologists were blinded to post-Baseline timepoints and all other patient information.

#### Secondary efficacy measures

##### Skeletal manifestations of HPP over time.

RGI-C scores and change from Baseline in Rickets Severity Scale (RSS) (20) scores were assessed at all study visits starting at Month 3. The RSS (20) is a 10-point scale [0 = absence of metaphyseal cupping and fraying (both characteristic of rickets) to 10 = severe rickets; maximum of four points for the wrists and six points for the knees] originally developed to assess the severity of nutritional rickets in the wrists and knees. Radiographs for determination of RSS score were read by a single independent rater who developed the RSS (Thomas D. Thacher, MD, Mayo Clinic, Rochester, MN). The percentage of responders (individual mean RGI-C score: ≥+2) at each study visit was also determined.

##### Respiratory status.

Respiratory status (including use and type of support) was assessed at Screening, Baseline, and all subsequent study visits.

##### Growth.

Length/height, weight, and head circumference were recorded during physical examinations at required study visits to assess changes in growth. Length/height and weight *z* scores were assigned based on the Centers for Disease Control and Prevention growth charts for age- and sex-matched healthy infants and children (21). Head circumference *z* scores were calculated using World Health Organization formulae (22).

##### Ventilator-free and overall survival.

Ventilator-free survival was assessed with the occurrence of death and ventilatory support [continuous positive airway pressure (CPAP), bilevel or biphasic positive airway pressure, or mechanical ventilation (invasive ventilation via endotracheal intubation or tracheostomy)]. Supplemental oxygen was considered respiratory but not ventilatory support. Survival was monitored throughout the study.

#### Other measures

Blood samples were collected to assess serum ALP activity, plasma PPi and PLP, and serum parathyroid hormone (PTH) concentrations at required study visits after an overnight fast and before study drug administration.

*ALPL* gene mutation analysis for patients not previously tested was performed by Connective Tissue Gene Tests (Allentown, PA).

### Safety and tolerability

Safety was assessed by routine reporting of adverse events (AEs), which included serious AEs, injection-site reactions (ISRs), and injection-associated reactions (IARs). ISRs were defined as treatment-emergent AEs (TEAEs) that were localized to the site of study drug administration, occurred at any time point after study drug initiation, and were assessed by the investigator as possibly, probably, or definitely related to study drug. IARs were defined as systemic signs, symptoms, or findings that occurred within 3 hours after study drug administration and were assessed by the investigator as possibly, probably, or definitely related to study drug. AEs of special interest included ectopic calcifications, lipodystrophy, craniosynostosis, and chronic hepatitis and were based on clinical review of observed AEs. Additional safety assessments included physical examinations, clinical laboratory tests (including calcium and magnesium), anti-asfotase alfa antibody levels (PPD Laboratories, LLC, Richmond, VA), fundoscopic eye examinations, and renal ultrasounds. The clinical significance of abnormal laboratory findings was judged by the investigator. Safety events reported after the study ended were not included.

### Responder analysis

A *post hoc* analysis compared Baseline characteristics of responders by radiography (RGI-C score: ≥+2) with those of nonresponders (score: <+2) at Year 1 of treatment.

### Statistical analysis

All efficacy and safety analyses were performed on the full analysis population (patients who received ≥1 dose of asfotase alfa). Some analyses were repeated on the per-protocol population (patients who received any asfotase alfa and had no major protocol deviations that could influence treatment effect). In general, continuous variables were summarized descriptively **(**data reported herein are median [minimum, maximum (min, max)] unless otherwise specified**)**, and categorical variables were summarized by counts and percentages of patients.

For the primary efficacy analysis (RGI-C scores at Month 6 and Year 1), a nonparametric Wilcoxon signed-rank test was used to determine whether the median RGI-C scores at Month 6 and Year 1 differed from 0. Missing values were imputed using last observation carried forward. Patients with no recorded post-Baseline values were assigned as having no change (score: 0).

Secondary efficacy analyses of RGI-C scores, percent of responders, and change from Baseline in RSS scores at each study visit were conducted in a manner similar to that used for the primary analysis; however, only observed data were used (no imputation). *P* values for length/height and weight *z* score were calculated *post hoc* using the nonparametric Wilcoxon signed-rank test comparing median change with zero. Pharmacodynamic and safety assessments are summarized descriptively. Ventilator-free survival and overall survival time were assessed using Kaplan-Meier methodology.

For the *post hoc* responder analysis, *P* values were calculated using the exact Wilcoxon rank-sum test for continuous variables and the Fisher’s exact test for categorical variables. Missing values at Year 1 were imputed with last observation carried forward. Patients with no post-Baseline data were assigned as having no change (score: 0) and considered nonresponders.

## Results

### Patients

**Figure 1.**
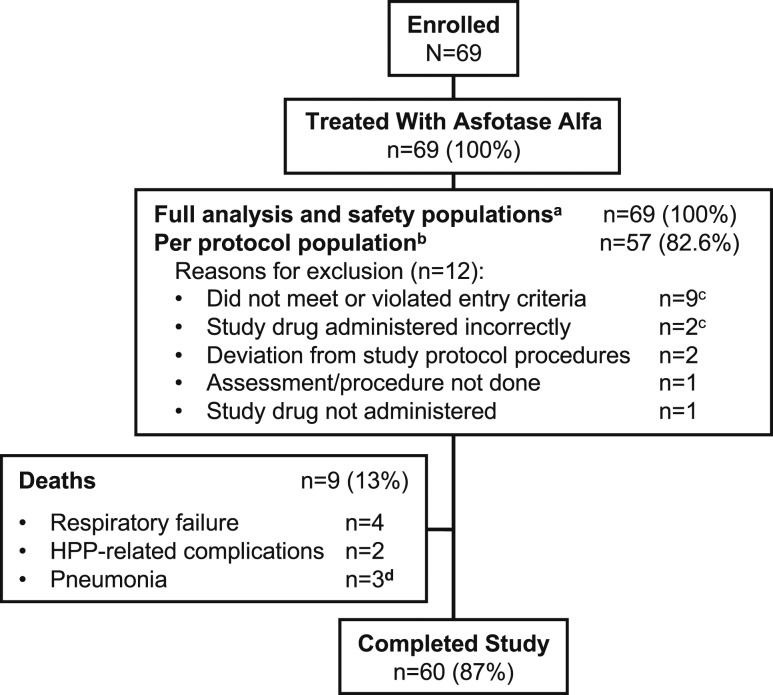
Patient disposition. ^a^All patients who received any asfotase alfa, regardless of whether they were lost to follow-up or dropped out of the study. ^b^All patients from the full analysis population who did not have any major protocol deviations deemed potentially to influence treatment effect. ^c^One patient did not meet the eligibility criteria and had incorrect administration of study drug. ^d^Considered possibly related to study drug treatment in one patient.

In total, 69 patients were enrolled from 22 sites in 12 countries and were included in the full analysis population, and 57 were included in the per-protocol population (Fig. 1). Patients were excluded from the per-protocol population if they did not meet entry criteria or violated entry criteria (n = 9), if they deviated from study protocol procedures (n = 2), if study drug was administered incorrectly (n = 2) or not at all (n = 1), or if an assessment/procedure was not done (n = 1; Fig. 1). The number of patients enrolled in each country was as follows: Australia (n = 1), Canada (n = 11), France (n = 5), Germany (n = 13), Italy (n = 2), Japan (n = 5), Russia (n = 1), Saudi Arabia (n = 1), Spain (n = 1), Turkey (n = 4), United Kingdom (n = 4), and United States (n = 21). Baseline demographic and clinical characteristics are summarized in Table 1.

**Table 1.** Baseline Demographic and Clinical Characteristics

Baseline Characteristic	Enrolled Patients, n = 69
Age at enrollment, month, median (min, max)	16.0 (0.3, 72.2)
Sex, n (%)	
Male	33 (48)
Race, n (%)	
White	54 (78)
Asian	7 (10)
Other	3 (4)
Unknown	5 (7)
Age at first signs of HPP, month, median (min, max)	1.0 (0, 5.5)
HPP-specific medical history, n (%)	
Abnormally shaped chest	58 (84)
History of respiratory compromise (up to and including respiratory failure)^*a*^	46 (67)
Seizures	17 (25)
Difficulty gaining weight, failure to thrive, and/or difficulty eating/swallowing	60 (87)
Hypercalcemia	61 (88)
Nephrocalcinosis	37 (54)
Fractures and/or delayed fracture healing	21 (30)
Length/height *z* score	n = 67
Median (min, max)	−2.7 (−10.0, 1.0)
Weight *z* score	n = 68
Median (min, max)	−2.5 (−24.0, 0)
RSS score	n = 67
Median (min, max)	4.0 (0, 10.0)
ALP, U/L (normal range: 60–370 U/L)^*b*^	n = 65
Median (min, max)	20 (18, 122)
PPi, μM (normal range: 1.3–5.7 µM)	n = 65
Median (min, max)	6.3 (2.7, 13.3)
PLP, ng/mL (normal range: 11.8–68.4 ng/mL)^*c*^	n = 60
Median (min, max)	521 (48, 24,600)
Calcium, mM [normal ranges: 2.25–2.74 mM (age: ≤2 years); 2.1–2.57 mM (age: >2 years)]	n = 65
Median (min, max)	2.6 (1.8, 4.0)

^a^ Respiratory compromise was defined as respiratory signs/symptoms that required institution of respiratory support measure(s), required medication(s) for management of symptom(s), and/or were associated with other respiratory complications (*e.g*., pneumonia, respiratory tract infection).

^b^ Normal range for ALP activity, per ARUP Laboratories (University of Utah, Salt Lake City, UT), varies by age: 0–30 days: 60–320 U/L; 1–11 months: 70–350 U/L; 1–3 years: 125–320 U/L; 4–6 years: 150–370 U/L. Normal range also varies by sex in patients older than 10 years of age.

^c^ Median (min, max) concentration for patients receiving vitamin B6 supplementation before dosing (n = 14) was 9960 (65, 24,600) ng/mL and for those patients not receiving vitamin B6 supplementation before dosing (n = 46) was 417 (48, 13,100) ng/mL.

### Dosing

Nearly all patients [67/69 (97%)] started asfotase alfa at 6 mg/kg/week; of the 67 patients, 64 (96%) received 2 mg/kg three times per week and three (4%) received 1 mg/kg six times per week. One patient started at 2 mg/kg seven times per week, and another started at 3 mg/kg three times per week. Doses were increased or decreased to 3 to 28 mg/kg/week for 17/69 (25%) patients to account for changes in body weight, to enhance the likelihood of a clinical response, or because of AEs and administration issues (volume and number of injections).

Overall, median treatment duration was 2.3 (0.02, 5.8) years. Of the 69 patients in the full analysis population, three (4%) received treatment for <3 months and 14 (20%) for ≥36 months.

### Primary efficacy measure

At Month 6 of treatment, the median (min, max) RGI-C score indicated significant improvement [+2.0 (−1.7, +3.0); *P* < 0.0001; n = 69]; most patients [40/69 (58%)] were considered responders, and six (9%) achieved a score of +3, indicating complete or near-complete healing of HPP-related skeletal manifestations. Results observed at Month 6 were consistent with those at Year 1 [+2.0 (−2.3, +3.0); *P* < 0.0001; n = 69]; 50/69 (72%) patients were considered responders, of which four (6%) achieved a score of +3. Results were similar in the per-protocol population (data not shown).

### Secondary efficacy measures

#### Skeletal manifestations of HPP over time

**Figure 2.**
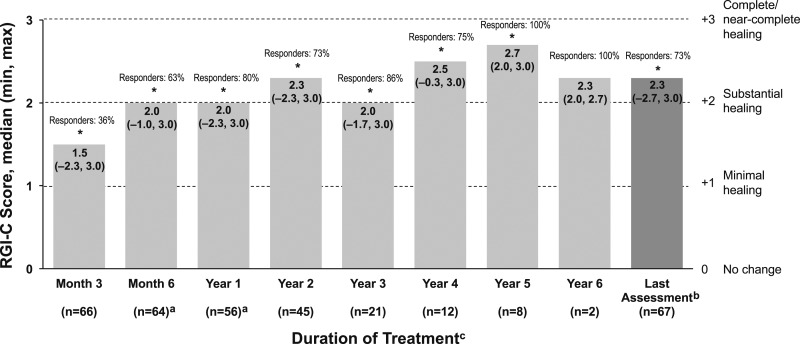
Median RGI-C scores over time in infants and children with HPP treated with asfotase alfa. Patients with RGI-C scores of ≥+2 were classified as responders. RGI-C scale: −3 = severe worsening of HPP-related skeletal manifestations; −2 = moderate worsening; −1 = minimal worsening; 0 = no change; +1 = minimal healing; +2 = substantial healing; +3 = complete or near-complete healing. ^a^Month 6 and Year 1 values differ from those in the text for the primary efficacy measure, as no imputation of missing values was performed. ^b^Last Assessment was defined as the latest post-Baseline assessment on treatment (within 5 days after end of treatment) with a nonmissing value for each patient; overall median (min, max) treatment duration was 2.3 (0.02, 5.8) years. ^c^All patients were included in the full analysis population; the decreasing n is a result of the number of patients on treatment at the end of the study or because assessments may not have been done at each time point. *P < 0.01 based on Wilcoxon signed-rank test comparing median change with zero.

**Figure 3.**
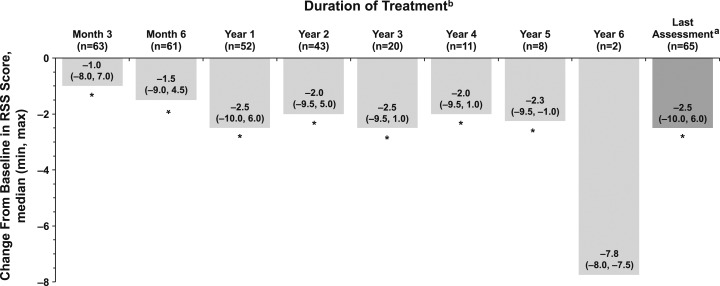
Median change from Baseline in RSS scores over time in infants and children with HPP treated with asfotase alfa. ^a^Last Assessment was defined as the latest post-Baseline assessment on treatment (within 5 days after end of treatment) with a nonmissing value for each patient; overall median (min, max) treatment duration was 2.3 (0.02, 5.8) years. ^b^All patients were included in the full analysis population; the decreasing n is a result of the number of patients on treatment at the end of study or because assessments may not have been done at each time point. *P < 0.05 based on Wilcoxon signed-rank test comparing median change with zero.

Preliminary patient-level radiographic outcomes have been published (14). Significant (*P* < 0.05) improvements in RGI-C score were observed at Months 3 and 6; Years 1, 2, 3, 4, and 5; and Last Assessment (Fig. 2). The proportion of patients classified as responders (RGI-C score ≥+2) increased during the study, from 36% (24/66 patients) at Month 3% to 73% (49/67 patients) at Last Assessment. Consistent with RGI-C scores, RSS scores improved significantly (*P <* 0.05) from Baseline at Months 3 and 6; Years 1, 2, 3, 4, and 5; and Last Assessment (Fig. 3). Results were similar in the per-protocol population (data not shown).

#### Respiratory status

Of the 45/69 (65%) patients who did not require respiratory support at Baseline, 38 (84%) lived without support during the study and 43 (96%) did not require support at the Last Assessment; one patient was receiving supplemental oxygen at Year 4, and one was receiving CPAP at Month 6. Three patients developed the need for respiratory support after Baseline but were weaned before Last Assessment (by Month 9, Year 1.5, and Year 2.5). Of the 24/69 (35%) patients who required respiratory support at Baseline (including invasive mechanical ventilation, CPAP, or supplemental oxygen), 11 (46%) no longer required support at Last Assessment.

#### Growth

**Figure 4.**
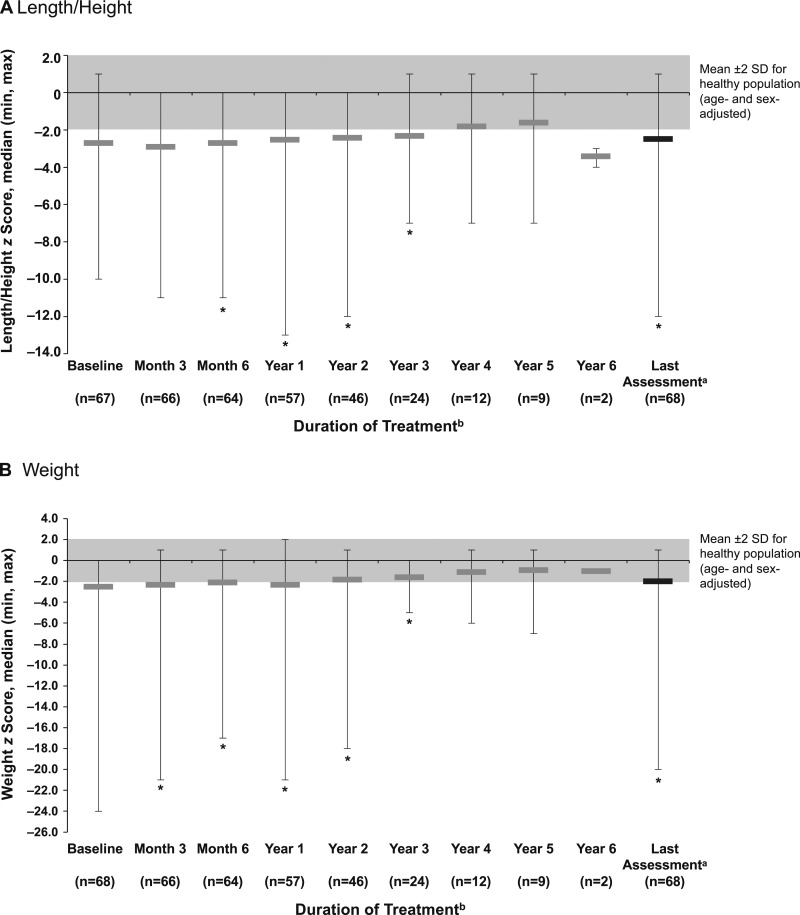
Median (min, max) *z* scores for (A) length/height and (B) weight over time in infants and children with HPP treated with asfotase alfa. ^a^Last Assessment was defined as the latest post-Baseline assessment on treatment (within 5 days after the end of the study treatment) with a nonmissing value for each patient; overall median treatment duration was 2.3 (0.02, 5.8) years. ^b^All patients were included in the full analysis population; the decreasing n is a result of the number of patients on treatment at the end of the study or because assessments may not have been done at each time point. *P < 0.05 based on Wilcoxon signed-rank test comparing median change with zero.

Length/height and weight *z* score generally improved over time (Fig. 4). Change from Baseline at Last Assessment was significant for both length/height [0.5 (−4, 4); n = 66; *P* = 0.0025] and weight [1.0 (−5, 6); n = 67; *P* = 0.0001] *z* score. Baseline head circumference *z* score was −1.0 (−4, 4; n = 56); change from Baseline was 0.1 (−2, 3; n = 47) at Month 6 and 0.2 (−3, 7; n = 55) at Last Assessment.

#### Ventilator-free and overall survival

Thirty-eight of the 45 patients (84%) who were not receiving respiratory support at Baseline remained ventilator free. The Kaplan-Meier estimate of the ventilator-free survival rate at Year 6 for these patients was 84%. Among all 69 patients, the Kaplan-Meier estimate of the overall survival rate at Year 6 was 80%. Survival outcomes for patients in this study were also included in a published analysis that pooled these data with those of a separate study (14).

### Other measures

Median (min, max) ALP activity increased from 20 (18, 122) U/L at Baseline (n = 65) to 3761 (272, 11,910) U/L after 3 weeks of treatment (n = 61) and continued to increase through Year 1 [6742 (1315, 20,041) U/L; n = 49]. ALP remained elevated throughout treatment, as expected with asfotase alfa treatment. Median (min, max) PPi concentration, which was elevated at Baseline [6.3 (2.7, 13.3) µM; n = 65], decreased to within reference range (1.3–5.7 µM) at Week 6 [3.9 (0.8, 39.2) µM; n=62] and remained within reference range throughout the study. Likewise, median (min, max) PLP concentration decreased from Baseline [521 (48, 24,600) ng/mL; n = 60] to within reference range (11.8 to 68.4 ng/mL) at Week 6 [44 (6, 4590) ng/mL; n=57] and remained within reference range through Year 5. PTH levels were 1.2 (0.6, 6.7; n = 48) pM at Baseline, 1.7 (0.6, 45.9; n = 52) pM at Month 6 and 2.2 (0.6, 10.1; n = 66) pM at Last Assessment.

Sixty-two patients had *ALPL* mutation analysis results; 44 patients were compound heterozygous for two pathogenic mutations, nine were homozygous for the same mutant allele, and nine had only one mutation identified, consistent with a dominant-negative effect.

### Safety and tolerability

All patients experienced at least one TEAE. Table 2 summarizes the most common TEAEs occurring in ≥20% of patients, regardless of the relationship to study drug. Most TEAEs were mild [2125/3052 (70%)] or moderate [728/3052 (24%)] in severity and assessed by the investigator as unrelated to study drug [2409/3052 (79%)]. The most common TEAEs assessed as related to study drug were ISRs [593/643 (92%)] and IARs [11/643 (2%)], which occurred in 43 and six patients, respectively. The most common ISRs were injection-site erythema [33/69 (48%)], discoloration [12/69 (17%)], induration [11/69 (16%)], and hematoma [10/69 (14%)]. IARs consisted of pyrexia [4/69 (6%)], chills [1/69 (1%)], injection-site rash [1/69 (1%)], anaphylactoid reaction [1/69 (1%)], drug hypersensitivity [1/69 (1%)], and papular rash [1/69 (1%)]. The IARs of anaphylactoid reaction (categorized as Stage 1 anaphylactic shock) and drug hypersensitivity were considered serious; neither patient had received pretreatment with medications to manage IARs. Both events resulted in interruption of asfotase alfa administration; the anaphylactoid reaction was treated with an IV electrolyte solution, and no treatment was given for the event of drug hypersensitivity. Both events resolved, and treatment with asfotase alfa was restarted without further occurrences.

**Table 2.** TEAEs Occurring in >20% of Patients—Safety Analysis Population (n=69)

TEAE^*a*^	Patients, n (%)
Pyrexia	47 (68)
Tooth loss	41 (59)
Injection-site erythema	33 (48)
Vomiting	31 (45)
Diarrhea	20 (29)
Craniosynostosis	19 (28)
Upper respiratory tract infection	19 (28)
Nasopharyngitis	18 (26)
Gastroenteritis	17 (25)
Cough	17 (25)
Respiratory tract infection	16 (23)
Constipation	16 (23)
Pneumonia	14 (20)

^a^ AEs coded using MedDRA Version 13.0 (Medical Dictionary for Regulatory Activities, McLean, VA).

Lipodystrophy was reported in 5/69 (7%) patients and was mild or moderate in severity and assessed as probably related or related to study drug. Eight patients [8/69 (12%)] had ectopic calcification findings on an eye examination, which were identified as TEAEs in two patients. Both events involved corneal deposits, were considered unrelated to study drug, did not interfere with vision, and resolved at Last Assessment. Nephrocalcinosis was reported in 46/69 (67%) patients and was present at Baseline in all but six patients. Five patients had nephrocalcinosis reported as a TEAE. An additional seven had eight TEAEs that were not recorded as ectopic calcifications or nephrocalcinosis but were considered as such upon medical review. Renal function remained normal in all patients.

A total of 28/69 (41%) patients experienced 46 events related to craniosynostosis (onset after start of treatment: 1 to 1851 days); all but three events were assessed as unlikely related or unrelated to study drug, and all but seven were mild or moderate in severity. Two patients required surgical treatment.

Twenty-two events related to chronic hepatitis were reported in 13/69 (19%) patients. All were mild or moderate in severity. Hepatomegaly in one patient was assessed as possibly related to study drug treatment. A serious AE of increased hepatic enzymes in another patient was moderate in severity and assessed as unlikely related to study drug.

A total of 50/69 (72%) patients experienced 297 serious AEs, most of which [286 (96%)] were assessed by the investigator as unlikely related or unrelated to the study drug. Of the 11 serious AEs considered treatment related, seven were ISRs or IARs in three patients. The remaining four occurred in three patients: craniosynostosis (n = 1), pneumonia resulting in study-drug withdrawal (n = 1), and Arnold-Chiari type 1 malformation and syringomyelia (n = 1).

In total, nine (13%) patients died. The causes of death in six patients were respiratory failure and cerebral death (following findings of hypoxia-induced lesions/encephalopathy 1 week before death); HPP-related complications; severe respiratory failure; cardiopulmonary arrest; severe cardiopulmonary insufficiency; and transtentorial and cerebellar tonsillar herniation as a result of cerebral edema related to severe HPP. Three patients died of pneumonia; in one patient, pneumonia was considered possibly related to asfotase alfa treatment.

Calcium and magnesium levels were of particular interest in this study population based on their role in bone formation, strength, and rigidity. The mean calcium level was within normal limits at Baseline [mean (SD): 2.6 (0.3) mM], and only small fluctuations were observed over the course of the study. Post-Baseline changes in calcium levels were considered clinically significant by the investigator in six patients; three patients had elevated levels [3.6 mM at Week 3 (n = 1); 3.6 mM at Week 3 and 3.3 mM at Week 6 (n = 1); and 3.1 mM at Week 120 (n = 1)], two of whom had elevated levels at Baseline, and three had decreased levels [2.1 mM at Month 3 (n = 1; age at Baseline: 217 weeks); 2.0 mM at Month 9 (n = 1; age at Baseline: 21 weeks); and 2.2 mM at Year 1 (n = 1; age at Baseline: 4 weeks)]. Magnesium levels remained generally within normal range; one patient had a clinically significant low level at Month 3 (0.5 mM) that was normalized at Months 6 and 9 (Last Assessment).

Twelve patients had clinically significant abnormal hematology findings. Five had low hematocrit and hemoglobin levels, five had high leukocyte or lymphocyte counts, one had low neutrophil count, and one had high blasts. For most patients, hematology findings returned to normal or were no longer considered clinically significant at the patient’s Last Assessment; one patient had clinically significant low hematocrit, hemoglobin, and erythrocyte levels at Years 3.5 and 4 (Last Assessment).

### Anti-asfotase alfa antibody levels

In total, 60/68 (88%) patients tested positive for anti-asfotase alfa antibodies during the study (maximum titer: 2048); 40 (67%) of these patients tested positive for neutralizing antibodies (NAbs). Six patients tested positive for anti-asfotase alfa antibodies at Baseline, one of whom tested negative at all subsequent assessments. The median (min, max) positive NAb titer at Last Assessment, measured as percent inhibition, was 7.7% (4.5, 92.6). Median (min, max) time to detection of first post-Baseline NAb titer was 168.5 (20, 1359) days. No clear relationship was found between the presence of anti-asfotase alfa antibodies and AEs nor were any AEs suggestive of immune mediation or tachyphylaxis.

### RGI-C responders

Results of the *post hoc* comparison between RGI-C responders (individual mean ≥+2 at Year 1) and nonresponders at Week 48 are summarized in Table 3. Of the 69 patients in this study, 50 (72.5%) had an RGI-C score of ≥+2 at Year 1. Nineteen patients did not achieve an RGI-C score of ≥+2 at Year 1; median RGI-C score for these nonresponders was 0.67 at Year 1. Seventeen of these nonresponders had a last overall on-treatment assessment, and of these, five achieved an RGI-C score of ≥+2 by ∼2.3 years. In this same timeframe, an additional four nonresponders achieved an RGI-C score between +1 and <+2. The remaining eight nonresponders had RGI-C scores <+1 at Last Assessment.

**Table 3.** Comparison of Baseline Characteristics and Outcomes in Radiographic Responders vs Nonresponders^*a*^—Full Analysis Population

	Responders (n = 50)^*b*^	Nonresponders (n = 19)^*b*^	*P* Value
**Disposition**			
Completed study, n (%)	47 (94)	13 (68)	0.0105
Discontinued, n			
Withdrawal by parents	1	2	
AE	2	4	
**Characteristics**			
Age at enrollment, month, median (min, max)	21.0 (0, 71.4)	8.9 (0.4, 71.7)	0.2318
Male, n (%)	25 (50)	8 (42)	0.5997
Age at HPP onset, month, median (min, max)	1.0 (0, 5.5)	1.0 (0, 5.0)	0.1164
Time from HPP diagnosis to treatment, month, Median (min, max)	20.3 (0, 67.8)	8.1 (0.3, 67.3)	0.3753
Weight *z* score	n = 49	n = 19	
Median (min, max)	−2.3 (−7.8, −0.04)	−2.7 (−23.8, −0.3)	0.3930
Length *z* score	n = 48	n = 19	
Median (min, max)	−2.6 (−8.3, 0.9)	−3.5 (−10.1, −0.3)	0.0345
Chest circumference, cm	n = 47	n = 19	
Median (min, max)	42.5 (32.0, 56.0)	37.0 (27.5, 51.5)	0.0261
Race, n (%)			
Asian	5 (10.0)	2 (10.5)	1.0000
White	38 (76.0)	16 (84.2)	
Multiple	1 (2.0)	0	
Other	2 (4.0)	0	
Unknown/not reported	4 (8.0)	1 (5.3)	
**Laboratory parameters, median (min, max)**			
ALP, U/L	n = 47	n = 18	
	23 (18, 122)	18 (18, 50)	0.0513
Calcium, mM	n = 47	n = 18	
	2.5 (1.8, 3.6)	2.6 (2.3, 4.0)	0.0204
PLP, ng/mL	n = 43	n = 17	
	429.0 (65.1, 24,100.0)	1300.0 (47.5, 24,600.0)	0.0403
PPi, µM	n = 46	n = 19	
	5.9 (2.7, 12.5)	7.1 (3.6, 13.3)	0.0427
Magnesium, mM	n = 47	n = 18	
	0.9 (0.6, 1.2)	0.9 (0.6, 1.1)	0.6433
Phosphate, mM	n = 47	n = 18	
	2.0 (1.2, 2.5)	2.0 (0.9, 2.7)	0.7918
PTH, pM	n = 34	n = 14	
	1.5 (0.6, 5.4)	0.6 (0.6, 6.7)	0.7302
***ALPL* gene polymorphism, n (%)**			0.6634
Compound heterozygous	31/45 (69)	13/17 (76)	
Dominant heterozygous	8/45 (18)	1/17 (6)	
Homozygous	6/45 (13)	3/17 (18)	
**Disease characteristics, n (%)**			
Respiratory compromise	33 (66)	12 (63)	1.000
Rachitic chest	49 (98)	17 (90)	0.1817
B6-responsive seizures	11 (22)	8 (42)	0.1320
Abnormally shaped chest	39 (78)	13 (68)	0.5327
High serum calcium	35 (70)	14 (74)	1.000
Nephrocalcinosis	25 (50)	12 (63)	0.4208
**Mean Baseline, mean RSS (SD)**	4.7 (3.1)	4.8 (3.5)	0.9833
**Respiratory characteristics**			
Fraction of inspired oxygen, %	n = 12	n = 7	
Median (min, max)	29.0 (21.0, 50.0)	25.0 (21.0, 54.0)	0.3500
Inspiratory pressure, cm H_2_O	n = 8	n = 5	
Median (min, max)	25.5 (0.4, 31.0)	24.0 (15.0, 32.0)	0.6601
Expiratory pressure, cm H_2_O	n = 9	n = 7	
Median (min, max)	7.0 (5.0, 12.0)	6.0 (5.0, 8.0)	0.2726
Respiratory support type, n (%)			
No support	35 (70)	10 (53)	0.5079
Mechanical ventilation	8 (16)	5 (26)	
Supplemental oxygen	4 (8)	2 (11)	
CPAP	2 (4)	2 (11)	
Other	1 (2)	0	
Respiratory support duration, hour	n = 14	n = 8	
Median (min, max)	24 (8, 24)	24 (24, 24)	0.4497
**Treatment exposure**			
Dosing frequency, n (%)			0.6642
Always received three times/week	46 (92)	17 (90)	
Ever received greater than three times/week	4 (8)	2 (11)	
Last dose received, mg/kg/week, median (min, max)	4.0 (2, 12)	5.7 (2, 11)	0.2924
Treatment duration, year, median (min, max)	2.3 (0.6, 5.8)	1.9 (0.02, 4.2)	0.1342
**ADA/NAbs**			
Positive ADA status, n/N (%)			
Month 6	34/48 (71)	12/15 (80)	0.7400
Year 1	28/44 (64)	9/10 (90)	0.1411
Ever	45/50 (90)	15/18 (83)	0.4279
Last Assessment	35/50 (70)	14/18 (78)	0.7603
Positive NAb status, n/N (%)			
Month 6	10/33 (30)	6/12 (50)	0.2963
Year 1	8/28 (29)	5/9 (56)	0.2293
Ever	29/45 (64)	11/15 (73)	0.7529
Last Assessment	9/45 (20)	9/15 (60)	0.0074
NAb by percent inhibition			
Month 6	n = 33	n = 12	
Median (min, max)	2.4 (−1.7, 90.3)	5.4 (−0.5, 88.5)	0.1820
Year 1	n = 3	n = 12	
Median (min, max)	2.5 (−6.2, 63.5)	4.7 (−3.5, 93.8)	0.3046
**Death**			
Number of deaths on study, n (%)	2 (4)	7 (37)	0.0012
**RGI-C scores**			
RGI-C score at Last Assessment	n = 50	n = 17	
Median (min, max)	+2.7 (−2.7, +3.0)	+1.0 (−1.7, +2.7)	<0.0001
Category, n (%)			
−3 to <−2	2/50 (4)	0/17	
−2 to <−1	0/50	1/17 (6)	
−1 to <0	1/50 (2)	4/17 (24)	
0 to <+1	0/50	3/17 (18)	
+1 to <+2	3/50 (6)	4/17 (24)	
+2 to <+3	40/50 (80)	5/17 (29)	
+3	4/50 (8)	0/17	

Abbreviation: ADA, antidrug antibodies.

^a^ RGI-C responders: mean score ≥+2 at Year 1.

^b^ Percentages of patients are calculated using the total numbers of responders (n=50) and nonresponders (n=19) in the full analysis population unless otherwise noted.

A greater proportion of responders than nonresponders completed the study (94% vs 68%, respectively; *P =* 0.0105), with fewer deaths (4% vs 37%, respectively; *P* = 0.0012). Compared with responders, nonresponders also had higher serum calcium (*P* = 0.0204), plasma PLP (*P* = 0.0403), and plasma PPi concentrations (*P* = 0.0427) at Baseline. Nonresponders had lower length/height *z* scores (*P* = 0.0345) and smaller chest circumferences (*P* = 0.0261) at Baseline than responders. Correlation between Baseline ALP activity and RGI-C score at Year 1 was moderate in nonresponders (Pearson correlation coefficient: 0.5468; *P* = 0.0189) and weak in responders (0.2008; *P* = 0.1759). There was no statistical difference between responders and nonresponders in anti-asfotase alfa antibody status during the study. Positive NAb status was not statistically significantly different between responders and nonresponders at Month 6 or at Year 1, but the percentage of nonresponders positive for NAbs was significantly higher (9/15; 60%) than the percentage of responders (9/45; 20%; *P* = 0.0047) at Last Assessment.

## Discussion

This open-label study of asfotase alfa treatment in infants and young children with a follow-up period of up to 6 years is the largest prospective clinical study of HPP to date. The enrolled population of 69 children with severe HPP is exceptionally large for a study of a rare disease such as HPP. This study assessed therapy response, showing that asfotase alfa was efficacious and safe in the majority of children who had onset of severe HPP signs/symptoms before age 6 months. Improvements in skeletal manifestations, respiratory support, and growth were observed during treatment with asfotase alfa within 6 months and were sustained for a median of 2.3 years and up to 6 years of treatment. Radiographic nonresponders at Year 1 generally had more severe disease at Baseline and a higher incidence of positive NAb status at the study end than responders.

The primary efficacy measures at Month 6 and Year 1 were met, showing substantial improvements in HPP-related skeletal manifestations. Moreover, 58% of patients were considered responders at Month 6 and 72% at Year 1, indicating substantial healing of skeletal manifestations. Improvements in respiratory status were also observed; nearly one-half (46%) of the patients who started the study requiring respiratory support were able to forego support by Last Assessment, and 89% who were free of respiratory support at Baseline remained so over the study. These results are consistent with those of previous smaller studies in infants and young children (n = 11; age: 2 weeks to 3 years) and in Japanese patients with HPP [n = 13; median (min, max) age at Baseline: 91 days (0 days, 34 years)] (17, 18, 23).

Nine deaths occurred in this study, mostly attributed to underlying HPP disease. The majority of deaths (78%) were radiographic nonresponders. One death, which was attributed to pneumonia, was considered by the investigator to be possibly related to asfotase alfa. In a prior survival analysis, which included data from some patients in the current study and other asfotase alfa studies, treatment significantly (*P* < 0.0001) improved survival among patients with perinatal and infantile HPP; survival was 95% at age 1 year and 84% at age 5 years but only 42% and 27%, respectively, among historical controls (14).

Asfotase alfa was generally well tolerated, with an overall safety profile consistent with that observed previously (14, 17, 23). All patients experienced at least one TEAE; most TEAEs were mild or moderate in severity and assessed as unrelated to study drug. Less than 10% of patients experienced serious AEs that were considered treatment related. The majority (64%) of treatment-related serious AEs were ISRs or IARs. Clinicians should be aware of strategies to minimize or prevent ISRs, such as proper injection technique, as well as management options for reactions requiring treatment (24). Post-Baseline elevated calcium levels were considered clinically significant in three patients, two of whom had clinically significant elevations at Baseline. Three patients had clinically significant hypocalcemia during the study. Previous reports discussed the role of dietary calcium restriction in the management of HPP before asfotase alfa became available, as many patients have a history of hypercalcemia (17, 23). The improved skeletal mineralization associated with asfotase alfa increases calcium intake requirements (*i.e.*, hungry bone syndrome). Hence, the supplying of sufficient dietary calcium and monitoring of serum calcium and PTH during initial treatment are essential (24). The monitoring of urine calcium in relation to serum calcium and PTH is also important in the guidance of calcium requirements.

Anti-asfotase alfa antibodies were identified in 88% of patients during this study, and 67% of these patients tested positive for NAbs. Development of antidrug antibodies has been documented in previous clinical studies of asfotase alfa, with no apparent impact on clinical outcomes (17, 25). These data reflect small sample sizes (9 to 12 patients) and a short duration of treatment exposure (up to 1 year). The current study, which includes the largest population to date of asfotase alfa-treated patients, with a median (min, max) treatment duration of 2.3 (0.02, 5.8) years, showed no clear relationship between the presence of these antibodies and AEs. A *post hoc* analysis suggests that NAb-positive status may be associated with a slow radiographic response, as assessed by the RGI-C at Year 1. Unfortunately, antidrug antibody testing is not yet commercially available to identify patients who may not be responding to treatment because of the presence of NAbs. The development of NAbs could be associated with the severity of the disease, as has been observed in other conditions, such as infantile-onset Pompe disease (26), but to date, the limited data available do not allow us to draw firm conclusions. A recent case report found that the development of NAbs (percent inhibition: 40.4%) in a patient with HPP treated with asfotase alfa for 2.5 years was associated with loss of efficacy and that immune-tolerance induction therapy and a 1-month discontinuation of asfotase alfa successfully restored treatment efficacy within 6 months (27). Currently, antidrug antibody testing is available for patients enrolled in the Global HPP Registry (NCT02306720; EUPAS13514), which will also allow for the collection of data to understand further the impact of NAbs on the efficacy and safety of asfotase alfa.

Generally, RGI-C nonresponders at Year 1 represent a subgroup of patients with more severe HPP at Baseline, evidenced by narrower chest walls (*i.e.*, smaller chest circumference), greater requirements for mechanical ventilation, and a higher proportion of deaths. Levels of TNSALP substrates (PPi and PLP) and serum calcium were also higher in nonresponders. These characteristics should be considered when determining dosing for patients with severe perinatal HPP. It should also be considered that patients with severe disease may have a delayed response to treatment or, in some cases, a limited response to treatment as a result of serious pre-existing complications of HPP, such as severe hypomineralization or lung hypoplasia.

A limitation of this study was the heterogeneity of study patients, as some patients had life-threatening perinatal disease. In addition, treatment was initiated at various ages, making comparisons difficult. Statistical analyses also did not correct for multiplicity or confounding variables. Lastly, in our *post hoc* responder analysis, although there were some patients who had minimal radiographic improvements, as a result of our strict definition of responders (RGI-C score ≥+2), these patients were included in the nonresponder group. Accordingly, nine patients in the nonresponder group, included based on their RGI-C scores at Year 1, had scores ≥+1 to <+3 at Last Assessment, indicating minimal or substantial healing.

## Conclusions

Most infants and young children with HPP treated with asfotase alfa showed sustained improvements in HPP-related skeletal manifestations, respiratory function, and growth. Asfotase alfa was generally well tolerated. A subgroup of the study patients with very severe disease at Baseline was classified here as radiographic nonresponders. These patients should be closely monitored for a therapeutic response, with dose adjustments and additional therapeutic measures considered as necessary.

## Abbreviations:

AE: adverse event
ALP: alkaline phosphatase
CPAP: continuous positive airway pressure
HPP: hypophosphatasia
IAR: injection-associated reaction
ISR: injection-site reaction
max: maximum
min: minimum
NAb: neutralizing antibody
PLP: pyridoxal 5′-phosphate
PPi: inorganic pyrophosphate
PTH: parathyroid hormone
RGI-C: Radiographic Global Impression of Change
RSS: Rickets Severity Scale
TEAE: treatment-emergent adverse event
TNSALP: tissue-nonspecific isoenzyme of alkaline phosphatase
